# The effect of Saqez (*Pistacia atlantica*) ointment on nipple fissure improvement in breastfeeding women during one-month follow-up

**Published:** 2017

**Authors:** Nayereh As’adi, Nourossadat Kariman, Faraz Mojab, Mohamad Amin Pourhoseingholi

**Affiliations:** 1 *Student Research Committee, School of Nursing and Midwifery, Shahid Beheshti University of Medical Sciences, Tehran, Iran*; 2 *Research Center for Midwifery and Reproductive Health, School of Nursing and Midwifery, Shahid Beheshti University of Medical Sciences, Tehran, Iran*; 3 *Department of Pharmacognosy, School of Pharmacy, Shahid Beheshti University of Medical Sciences, Tehran, Iran*; 4 *Liver Department, Research Center for Gastroenterology and Liver Disease, Shahid Beheshti University of Medical Sciences, Tehran, Iran*

**Keywords:** Nipple fissure, Pain, Sagez ointment, Pistacia atlantica, Breast milk

## Abstract

**Objective:**

Painful nipple fissure is a troublesome problem for breastfeeding mothers. This study was conducted to evaluate the effect of saqez (*Pistacia atlantica*) ointment on the improvement of nipple fissure in breastfeeding women during one-month follow-up.

**Materials and Methods:**

This randomized controlled clinical trial was conducted on 100 eligible women who visited the selected health centers affiliated to Shahid Beheshti University of Medical Sciences, Tehran, Iran, from July 2015 to December 2015 during their postpartum period. A total of 100 subjects were randomly divided into two equal groups of 50 women grouped as saqez ointment group and breast milk group, and followed-up for one month. Both groups received face-to-face instructions on breastfeeding techniques. For severity of nipple fissure, Storr scale and to measure the intensity of pain, visual analog scale (VAS) were used.

**Results:**

The results showed that the two groups were matched in terms of demographic and obstetric characteristics. Mean of nipple fissure severity in ointment group (42.62) was lower than that of the control group (48.02), that was significantly different between the two groups (p=0.047). In addition, Mean nipple pain intensity in ointment group (40.57) was lower than that of the control group (49.81), but there was no significant difference between the two groups (p=0.056).

**Conclusion:**

The present study showed that saqez ointment was more effective than breast milk in healing and controlling nipple fissures during one-month follow-up, without resulting in any side effects.

## Introduction

Breastfeeding is universally considered as the best method of infant nutrition. Breast milk contains almost all the necessary nutrients, immunological components and growth factors that a term infant needs (Heller et al., 2012 ▶). World Health Organization (WHO) recommends breastfeeding for at least the first 6 months of life (World Health Organization, 2016 ▶).

But, painful sore nipples are a troublesome problem for mothers and cause early cessation of breastfeeding (Oğuz et al., 2014 ▶). Nipple trauma is defined as the presence of pain in the nipple wound caused by sucking which is graded from mild to severe with physical damage (fissure, wound, bleeding, edema, erythema and blisters) associated with breastfeeding (Ahmed et al., 2015 ▶). This soreness usually occurs between days 3 and 7 postpartum and in some mothers may persist to 6 weeks after delivery (Akbari et al., 2014). The occurrence rate of nipple soreness and nipple pain during breastfeeding is 11-96% (Marrazzu et al., 2015 ▶).

Nipple pain during breastfeeding is a cause of concern for mothers in the immediate postpartum period, (Kent et al., 2015 ▶). Nipple fissure is a threat for mothers, babies and societies. It is associated with reduced exclusive breastfeeding and high levels of maternal stress. In addition, any defect in the skin surface is prone to bacterial and fungal infection (Dennis et al., 2012 ▶, Shanazi et al., 2015 ▶). Many predisposing factors have been reported for nipple fissure. The prominent reason is incorrect breastfeeding technique such as incorrect positioning and poor latch-on (Chaves et al., 2012 ▶). There are two treatments for nipple soreness: dry wound healing (exposure to the sun and sun bathing) and moist healing (use of breastmilk, creams and suitable ointments) (Essa and Ebrahim, 2013 ▶). Currently, moisture is used for healing wounds, because moist environment creates epithelization, proliferation and epithelial migration on the surface of the wound during healing time. Current guidelines recommend the use of purified lanolin ointment and breast milk in the treatment of nipple fissures, based on moist wound healing principles (Marrazzu et al., 2015 ▶). However, effectiveness of these treatments is controversial (Marrazzu et al., 2015 ▶; Cadwell et al., 2004 ▶). Furthermore, healing time in breast milk-treated mothers is longer compared to other treatments (As̕adi et al., 2016 ▶).

None of the previous studies have proposed the most effective cure for nipple fissures. So, there is a great need for further and more precise studies (Buck et al., 2015 ▶, As'adi N, 2016 ▶). Recently, due to the failure of the existing treatments, researchers have been inclined to use herbal medicines (Abou-Dakn et al., 2011 ▶; Alamolhoda, 2014 ▶). 

Observations and experiences of the researcher showed that sagez ointment has been recommended by locals as an ideal and effective treatment for nipple fissure in West Azarbayjan Province of Iran.

Saqez (*Pistacia atlantica*, from the Anacardiaceae family), is an oleoresin obtained by scratching the trunks of Baneh trees. Saqez contains flavonoids and flavonoid glycosides, triterpenoids and phenolic compounds with strong antioxidant and antimicrobial properties and thus, cures wounds (Bozorgi et al., 2013 ▶, Tanideh et al., 2014 ▶). Also, saqez has antimicrobial activity against Gram-positive and –negative bacteria that are resistant to common antimicrobial treatments. In addition, it has antifungal and antiviral activity (Khodavaisy et al., 2016 ▶, Bozorgi et al., 2013 ▶). Saqez ointment has also been traditionally used for the treatment of peptic ulcer disease, swelled and cracked lips and also anal fissure. It has also been traditionally used as wound healing promoter in Kurdistan, Iran (Gourine et al., 2010 ▶; Haghdoost et al., 2013 ▶; Bahmani et al., 2015 ▶). 

Due to the importance of exclusive breastfeeding and a great need for the treatment of nipple fissures in the shortest time possible and because of wound healing properties of saqez ointment, the present study was conducted to determine the effect of saqez ointment on the improvement of nipple fissure in breastfeeding women during one-month follow-up. So far, no studies have been done to investigate the effect of saqez ointment on nipple fissures or pain.

## Materials and Methods

The study was approved by Shahid Beheshti School of Nursing and Midwifery: (SBMU2.REC.1394.10). Furthermore, it was registered at the Iranian Registry of Clinical Trials (IRCT 2015080723535N1).

This randomized controlled clinical trial was conducted on 100 eligible women referred to selected health centers affiliated to Shahid Beheshti University of Medical Sciences, Tehran, Iran from July 2015 to December 2015. The sample size of this study was estimated to be 100 participants with a potential sample loss of 10-15% in the follow-up stages. This sample size was calculated based on the results of previous studies (Alamolhoda et al., 2014 ▶ and Saeidi et al., 2015 ▶) with a confidence interval of 95%, the test power of 80%, α= 0.05, P1= the percentage of improvement in the Saqez group and P2= the percentage of improvement in the control group (Munro 2005 ▶). 

**Figure1:**
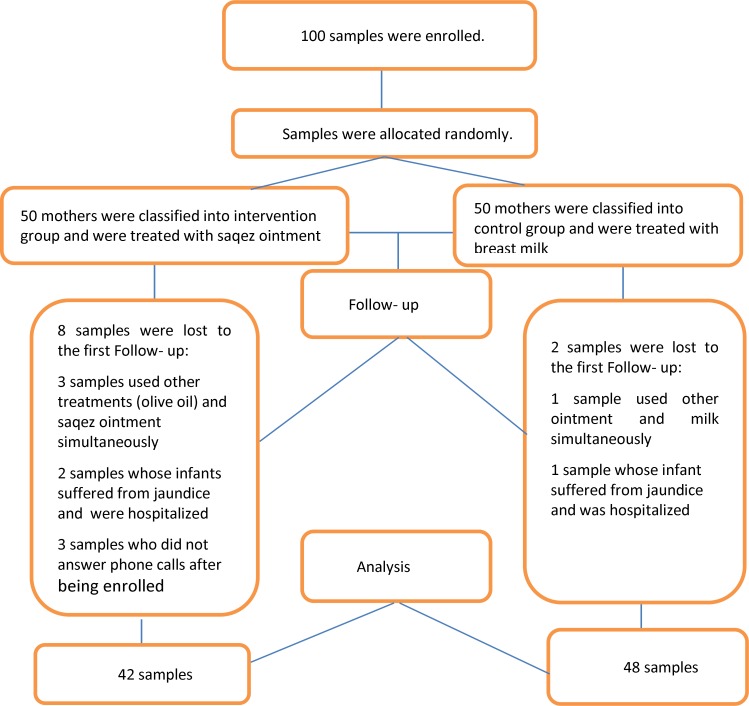
The CONSORT flow diagram of the trial

In this study, 100 eligible women were selected through purposive sampling and randomly divided into two equal groups of 50 saqez ointment and the control group, using Excel software. Ten individuals were excluded based on the exclusion criteria. Finally, we had 42 individuals in the sagez ointment group and 48 in the control group (Figure 1). Mothers who were over 18 years old, literate, and Iranian, had nipple fissure and pain and a minimum score of three on the Storr scale for assessing fissure, had a healthy term infant, had a singleton pregnancy, with a birth weight of 2500-4000 g, with exclusive breastfeeding, had no oral, palate or maxillofacial abnormalities in the newborn, no nipple abnormalities and had no history of allergy to saqez ointment, were included. The participants̕ level of pain during breastfeeding was measured using the pain scale and the severity of fissure was examined on the Storr scale. 

Nipple pain was assessed using Visual Analog Scale. This scale has 11 points; point zero was given for ‘no pain’ and the score of ten showed extremely severe pain. Previous studies have shown that the pain scale has adequate validity (Ferreira-Valente et al., 2011) and it has been used in several studies for the assessment of the severity of breast pain (McClellan et al., 2012 ▶). Farar et al. used the test-retest method to confirm the reliability of the pain scale and calculated its correlation coefficient as 0.83 (Farrar et al., 2008 ▶). In one study, Tafazoli et al. examined the parallel-forms reliability of the scale (Tafazoli, 2015 ▶).

Storr scale was used for assessing the severity of nipple fissure in this study. It has five degrees, from zero to four as follows: A painless nipple with normal color = 0, a slightly reddened nipple with pain at the beginning of breastfeeding (the first five to ten seconds) =1, a reddened nipple with pain at the beginning of and in the intervals between breastfeeding times = 2, a nipple beginning to develop fissure with pain at the beginning of and in the intervals between breastfeeding times=3 and a nipple fissure with pain at the beginning of and in the intervals between breastfeeding times =4. The Storr scale has been validated in 1988 by Storr using the content validity method and by measuring its Cronbach's alpha coefficient (Storr, 1988 ▶); the scale has also been confirmed for use in Iran by many studies by measuring its content validity (Tafazoli, 2015 ▶). 

The mothers who got infected with mastitis, abscess or fungal infection of the breast, used a breast pump or plastic nipple or used the recommended treatment less than three times per day or used other treatments and the recommended treatment simultaneously or did not answer the phone calls after being enrolled were excluded from this study. In addition, infants who used a pacifier or were fed with milk bottle, got sick and were hospitalized during the study and showed allergic reactions to saqez were excluded. On the day of admission, the researcher examined the newborns for oral thrush and potential oral and maxillofacial abnormalities and examined the mothers’ two nipples and recorded the severity of their nipple fissure and inflammation according to the Storr scale. Both groups then received face-to-face instructions on breastfeeding techniques and hygiene using a free educational pamphlet. The researcher then, asked each mother to breastfeed her newborn and assessed her breastfeeding technique and asked her to state her level of pain based on the pain scale. The demographic and obstetric questionnaire was then completed for the mothers and a checklist of side-effects and satisfaction with the treatment (whether or not she experienced any side effects at the site of the fissure, and whether or not she was happy with the treatment) was then given to the mothers and they were asked to complete and return the checklist to the researcher.

The control group was advised to apply two to three drops of their breast milk on nipple fissure and areola after each feeding. Saqez ointment was given to the intervention group at the beginning of the experiment and the mothers in this group learned to detect allergic reactions and then begin the treatment if there were no such reactions. Mothers were also advised to wash their hands immediately after each breastfeeding and to apply a fingertip of saqez ointment on their nipple fissure and areola three times per day.

The mothers were advised by the researcher to shower with warm water every day, keep their nipples dry and avoid using soap or other materials that could cause dryness of the nipple skin. The researcher then gave the mothers her phone number to contact at any time if they had any questions.

The mothers were visited again on the third day, the seventh day and also one month after the intervention. In these follow-ups, the mothers' breastfeeding technique was assessed again. Also, their level of pain during breastfeeding and intensity of their fissure were measured and recorded with the help of a research collaborator who was blinded to the groupings. In case of severe pain over the course of the study, the subjects were allowed to use analgesics, but had to record the type and number of analgesics taken and measure their severity of pain immediately before and half an hour after their use on the pain scale according to the instructions given.

The mothers who failed to achieve full recovery from nipple fissure and pain by the seventh day, were referred to a specialist. The effect of saqez ointment on nipple fissures and pain was conducted at two stages: one week post-treatment and after one month follow-up period (to determine the recurrence of nipple fissures and pain). The first stage of the study was presented in another article (As̕adi et al, 2017 ▶). 

**Components**

Saqez, is an oleoresin produced by scratching the trunks of Baneh trees; then, its moisture evaporates in the air and yields a white-yellow and semisolid substance (Bozorgi et al., 2013 ▶, Amin Gh, 2008 ▶). Ghee, is an oil obtained by heating animal butter (Saadatiyan et al., 2015 ▶). Beeswax (or yellow wax) is the wax obtained from honeycomb of the bee (Tyler VE et al.,1998 ▶). The ingredients of saqez ointment (saqez, ghee, and beeswax) were purchased from the herbal market in Urmia, Iran in July 2015. Then, Department of Pharmacognosy, School of Pharmacy, Shahid Beheshti University of Medical Sciences confirmed their scientific identity.

**Preparation of saqez ointment**

Ghee, saqez and beeswax (42%, 29% and 29%) were melted separately over a mild heat, then mixed by an electric mixer and poured into a jar (each jar contains 7 g of saqez, 10 g of ghee and 7 g of beeswax). 

**Statistical analysis**

Based on the results of previous studies, the sample size of this study was calculated to be 50 for each group, making a total of 100 participants, with a confidence interval of 95%, a type I error of 0.05, a type II error of 0.2 and a potential sample loss of 10-15% in the follow-up stages (Munro, 2005 ▶). The data were analyzed by SPSS-21. The descriptive assessment of the data and the demographic findings evaluation were presented as the mean and standard deviation, frequency tables and statistical charts. The comparison of the groups and inferential assessments were performed using the Cumulative Logit model, non-parametric Mann-Whitney test, independent t-test, Chi-square test and Fisher's exact test. The level of statistical significance was set at p<0.05.

## Results

The results obtained showed that the two groups were matched in terms of demographic and obstetric characteristics (Tables 1 and 2). Regarding the education, most of the participants in both groups 48 (52.9%) had academic education and 73 (80.9%) were housewives. In terms of economic status, the majority of the subjects in both groups 70 (78.9%) had low to middle income.

**Table 1:** Demographic Characteristics of the participants

**Groups** **variable**	**ointment group (42)**	**control group** **(48)**	**p-value**
**Maternal age (year)**	3.91± 30.02	4.64± 28.65	0.135
**Maternal weight (kg)**	10.84 ± 77.29	73.39±10.43	0.086
**Maternal height (cm)**	6.36 ± 162.62	6.01± 161.71	0.48
**Maternal BMI (kg/m2**	3.79 ± 29.29	4.31 ± 28.13	0.186
**Neonatal age (day)**	3.80± 5.38	9.65 ± 6.10	0.65
**Neonatal weight in birth (gr)**	335.22 ± 3240.24	423.8 ± 3137.2	0.209

**Table 2:** Obstetrical Characteristics of the participants

**Group** **variable**		**Ointment group (n=42)**		**Control group ** **(n=48)**		**p-value**
**Number of pregnancies**	1,2		36(85.7)		43(89.6)	0.578
	3,4		6 (14.3)		5 (10.4)	
**History of abortion**	positive		9 (21.4)		8 (16.7)	0.599
	negative		33(78.6)		40 (83.3)	
**Number of deliveries**	1,2		38 (90.5)		48 (100)	0.030
	3,4		4 (9.5)		0 (0)	
**Type of delivery**	vaginal		6 (14.3)		11 (22.9)	0.419
	C-section		36 (85.7)		37 (77.1)	

**Table 3:** A comparison between the ointment and control groups in terms of intensity of fissure

**group**	**Intensity of fissure**	**Mean rank**	**p-value** **(Wilcoxon test)**
**Ointment group** **(n=42)**	Before intervention	50.33	p<0.001
	1 month follow up	42.62	
**Control group** **(n=48)**	Before intervention	41.27	p<0.001
	After 1-month follow up	48.02	

**Table 4:** A comparison between the ointment and control groups in terms of intensity of pain

**group**	**Intensity of pain**	**Mean rank**	**p-value** **(Wilcoxon test)**
**Ointment group** **(n=42)**	Before intervention	54.77	p<0.001
	After 1-month follow up	40.57	
**Control group** **(n=48)**	Before intervention	37.39	p<0.001
	After 1-month follow up	49.81	

Table 3 shows that mean rank of nipple fissure severity before intervention, in the ointment and control groups were 50.33, 41.27, respectively and after one-month follow-up were 42.62 and 48.02, respectively. The results showed that there was a significant difference (p = 0.047) between the two groups in terms of nipple fissure severity after one-month follow-up compared to before-intervention data (Table 3). Table 4 compares the ointment and control group in terms of pain intensity. Mean rank of nipple pain intensity before intervention in the ointment and control group were 54.77 and 37.39, respectively and after one-month follow-up were 40.57and 49.81, respectively. However, there was no significant difference (p=0.056) between the two groups regarding nipple pain intensity after one-month follow- up (Table 4). Since the distribution of nipple fissure and pain intensity was not normal, non-parametric equivalent test was used. In the follow-up period, 1 patient (2.4%) in saqez ointment group and 17 patients (35.4%) in control group required further referral and treatment due to nipple fissure. Fisher's Exact test showed a significant difference between the two groups in terms of the need for further referral and treatment after the follow-up period (p<0.001). Thus, during one-month follow-up, saqez ointment was able to control and cure nipple fissures more than breast milk. In the present study, saqez ointment did not change the taste of milk and it did not have any significant side effects on the mother or baby. Both were assessed by using a checklist of side-effects and satisfaction with the treatment. 

## Discussion

Findings of the study showed that saqez ointment is more effective than breast milk in healing nipple fissure and reducing nipple pain in lactating women. In addition, it had more marked effects in controlling and curing nipple fissures compared to breast milk during one-month follow-up. The main reason for using saqez ointment and comparing it with the routine treatment (Expressed Breast Milk), was that saqez contains alpha-pinene, monoterpenoids and triterpenoids, which are responsible for its anti-inflammatory properties. The antimicrobial activity of saqez, especially against *Helicobacter pylori*, Gram-positive and Gram-negative bacteria and also its anti-fungal and antiviral properties are mostly due to the presence of alpha-pinene (Tanideh et al., 2014 ▶; Pourreza et al., 2008 ▶). Saqez has angiogenetic and wound-healing properties and has traditionally been used to dress wounds (Haghdoost et al., 2013 ▶).

The positive effect of ghee as the other ingredient of the ointment, on healing wounds, is due to the presence of saturated and unsaturated fatty acid compounds and vitamins E, A and D (Saadatiyan, 2015 ▶). Beeswax, the other constituent of the ointment, contains monoesters, di-esters, free fatty alcohols and free fatty acids. Thus, it is used as an emulsifier and softener in pharmaceutical and cosmetic products. (Asgarirad, 2004 ▶; Aeenechi, 1999 ▶). 

 Haghdoost et al. (2013) ▶ conducted a controlled study and showed positive dose-dependent effects of saqez on skin burns in rats due to fibroblast and platelet-derived growth factors and increased angiogenesis (Haghdoost et al., 2013 ▶). In a review study, Bahmani et al. (2015) ▶ confirmed the anti-inflammatory, antimicrobial, anti-fungal, antiviral, antioxidant and anti-cancer properties of saqez and described its uses in both traditional and medical treatment of skin wounds and cracks, which is consistent with the present findings (Bahmani et al., 2015 ▶).

In an experimental study, Saadatiyan et al. (2015) ▶ showed that the mixture of *Curcuma longa* L. and bovine ghee decreases stomach wound index, blood capillaries density and also increases mucosal layer thickness in the experimental stomach ulcers of rats. Probably, the bovine ghee, one of the constituent parts of saqez ointment, increases angiogenesis because of its vitamin E content, which is an important factor for the formation of new capillaries and thus, accelerates the healing process of the wounds (Saadatiyan et al., 2015 ▶)

Mohammadzadeh et al. (2005) ▶ conducted a randomized clinical trial and showed that healing time in lanolin-treated group was significantly longer than the breast milk (p=0.029) and the control group (p=0.028). Improvement of sore nipples in lanolin-treated group was 0% during days 1-3 and 55.4% during days 4-6 (Mohammadzadeh et al., 2005 ▶). In this study, healing rate in lanolin-treated subjects seems to be lower in comparison to saqez ointment. However, since there are differences in the design of the studies, diagnostic methods, duration of treatment, and assessment time, it is not possible to make an accurate comparison between topical methods. So, there is a need to conduct a new clinical trial to compare the effect of lanolin and saqez ointment. 

In this study, one-month follow-up indicated that saqez ointment is more effective than breast milk in healing nipple fissure and reducing nipple pain in breastfeeding women. Due to beneficial properties of saqez in the treating wounds and reducing the severity of pain with no side-effects on the mother or the baby, it can be recommended by the midwives, gynaecologists, nurses and health care providers as an inexpensive, effective and herbal cure for improving nipple fissures and reducing nipple pain. The limitation of the present study was that the subjects' response to pain varies markedly and cannot be controlled (Guyton, 2011 ▶).
